# Genome-Wide Characterization of HSP90 Gene Family in Cucumber and Their Potential Roles in Response to Abiotic and Biotic Stresses

**DOI:** 10.3389/fgene.2021.584886

**Published:** 2021-02-04

**Authors:** Kaijing Zhang, Shuaishuai He, Yihu Sui, Qinghai Gao, Shuangshuang Jia, Xiaomin Lu, Li Jia

**Affiliations:** ^1^College of Agriculture, Anhui Science and Technology University, Fengyang, China; ^2^Key Laboratory of Genetic Improvement and Ecophysiology of Horticultural Crop, Institute of Horticulture, Anhui Academy of Agricultural Sciences, Hefei, China

**Keywords:** cucumber, HSP90, abiotic stress, biotic stress, big data, expression profiles

## Abstract

Heat shock protein 90 (HSP90) possesses critical functions in plant developmental control and defense reactions. The HSP90 gene family has been studied in various plant species. However, the HSP90 gene family in cucumber has not been characterized in detail. In this study, a total of six HSP90 genes were identified from the cucumber genome, which were distributed to five chromosomes. Phylogenetic analysis divided the cucumber HSP90 genes into two groups. The structural characteristics of cucumber HSP90 members in the same group were similar but varied among different groups. Synteny analysis showed that only one cucumber HSP90 gene, Csa1G569290, was conservative, which was not collinear with any HSP90 gene in *Arabidopsis* and rice. The other five cucumber HSP90 genes were collinear with five *Arabidopsis* HSP90 genes and six rice HSP90 genes. Only one pair of paralogous genes in the cucumber HSP90 gene family, namely one pair of tandem duplication genes (Csa1G569270/Csa1G569290), was detected. The promoter analysis showed that the promoters of cucumber HSP90 genes contained hormone, stress, and development-related *cis*-elements. Tissue-specific expression analysis revealed that only one cucumber HSP90 gene Csa3G183950 was highly expressed in tendril but low or not expressed in other tissues, while the other five HSP90 genes were expressed in all tissues. Furthermore, the expression levels of cucumber HSP90 genes were differentially induced by temperature and photoperiod, gibberellin (GA), downy mildew, and powdery mildew stimuli. Two cucumber HSP90 genes, Csa1G569270 and Csa1G569290, were both differentially expressed in response to abiotic and biotic stresses, which means that these two HSP90 genes play important roles in the process of cucumber growth and development. These findings improve our understanding of cucumber HSP90 family genes and provide preliminary information for further studies of cucumber HSP90 gene functions in plant growth and development.

## Introduction

Plants are usually exposed to different kinds of environmental stress conditions during their growth and development. The primary environmental stresses included abiotic stresses such as high temperature, drought, chilling, salinity, and chemical pollutants and biotic stresses such as fungi, bacteria, viruses, and nematodes, which always cause cell injury and produce secondary stresses such as osmotic and oxidative stresses (Wang et al., 2004). In recent years, with the continuous development of global warming, high temperature stress has become one of the main factors affecting the normal growth and development of plants (Ahuja et al., 2010).

Heat shock proteins (HSPs) are a group of proteins induced by heat shock, which are widely distributed in various organisms from prokaryotes to eukaryotes (De Maio, 1999). Under the condition of high temperature stress, the organisms will activate and accumulate a large amount of the HSPs to participate in the heat shock response to maintain the stability of cells (Richter et al., 2010). Based on the molecular weight, HSPs are classified into five major families: sHSP, HSP60, HSP70, HSP90, and HSP100 (Wang et al., 2015; Muthusamy et al., 2017; Genest et al., 2019). HSP90 is a heat shock protein family with a molecular weight of about 90 kD, which is composed of three structural domains, including the N-terminal region containing ATP binding and hydrolysis sites, the middle region (M) and the C-terminal region containing dimerization regions (Rizzolo et al., 2014). HSP90 proteins are mainly distributed in cytoplasm, mitochondria, chloroplasts, and the endoplasmic reticulum (Ishiguro et al., 2002; Cao et al., 2003; Prassinos et al., 2008). HSP90 is an abundant and highly conserved molecular chaperone that is essential for viability in eukaryotes, which fulfills housekeeping functions on important biological processes such as signal transduction and cell cycle (Johnson and Brown, 2009).

The essential cellular functions of the molecular chaperone HSP90 have been intensively investigated in fungal and mammalian model systems (Sangster and Queitsch, 2005). The HSP90 genes have also been studied in some important plants, such as *Arabidopsis thaliana*, rice, *Populus*, tomato, pepper, and so on. In *Arabidopsis thaliana*, seven HSP90 family members were identified (Krishna and Gloor, 2001), which was the same number of HSP90 family members identified in tomato (Liu et al., 2014) and pepper (Wang et al., 2020). Nine and 10 HSP90 genes were identified in rice (Hu et al., 2009) and *Populus* (Zhang et al., 2013), respectively. Recently, the HSP90 gene family was also identified in *Nicotiana tabacum* (Song et al., 2019), *Aeluropus littoralis* (Hashemi-petroudi et al., 2019), *Hordeum vulgare* (Chaudhary et al., 2019), *Camellia sinensis* (Chen et al., 2018), and 44 plants (covering the stages from lower plants to higher plants, including algae, moss, ferns, gymnosperms, and angiosperms; Li et al., 2020). The diverse biological function of the HSP90 gene has also been well characterized in some plants. For example, in *Arabidopsis*, *AtHsp90.5* was important for chloroplast biogenesis and embryogenesis (Feng et al., 2014; Oh et al., 2014). In *Brassica napus*, HSP90 was found to play a role in the process of seed development and germination (Reddy et al., 1998). In cotton, HSP90 families played a crucial role in cotton fiber differentiation and development by maintaining cellular homeostasis (Sable et al., 2018). Plant HSP90 genes also played important roles in response to abiotic stresses. For instance, the HSP90-SGT1 chaperone system was required for the plant response to high temperature (Wang et al., 2016). The accumulation of HSP90 transcripts in *Brassica napus* was induced in response to the cold condition (Krishna et al., 1995). In rice, *OsHSP50.2* positively regulated drought stress tolerance probably by modulating ROS homeostasis and osmotic adjustment (Xiang et al., 2018). Overexpression of *AtHsp90.2*, *AtHsp90.5*, and *AtHsp90.7* in *Arabidopsis thaliana* enhanced plant sensitivity to salt and drought stresses (Song et al., 2009). Overexpression of *AtHsp90.3* in *Arabidopsis thaliana* impaired plant tolerance to heavy metal stress (Song et al., 2012). With the exception of HSP90 genes responding to abiotic stresses, HSP90 genes also played crucial roles in response to biotic stresses. *Arabidopsis* cytosolic HSP90 associated with and modulated the RPM1 disease resistance protein (Hubert et al., 2003). The suppression expression of *TaHsp90.2* or *TaHsp90.3* genes compromised the hypersensitive resistance response of wheat to stripe rust fungus (Wang et al., 2011). The above studies indicated that the HSP90 family genes are multifunctional in the life cycle of plants.

Cucumber (*Cucumis sativus* L.) is the first vegetable crop that finished the complete genome sequencing project (Huang et al., 2009), and is widely cultivated around the world [UN Food and Agriculture Organization Corporate Statistical Database (FAOSTAT), 2017]. With the high-quality genome information, a lot of gene families have been identified in cucumber, such as WRKY (Ling et al., 2011), MADS-box (Hu and Liu, 2012), NBS (Wan et al., 2013), bZIP (Baloglu et al., 2014), and so on. However, the identification of HSP90 gene family has not been reported so far, which largely limits the research of the biological function of HSP90 genes in cucumber. Only a few studies reported the functions of cucumber HSP genes, such as chitosan oligosaccharides treatment which resulted in upregulated HSP70 and HSP45.9 expression during cold storage (Ru et al., 2020) and ABA triggered the expression of HSP70 in an apoplastic H_2_O_2_-dependent manner under heat stress (Li et al., 2014). To the best of our knowledge, the functions of cucumber HSP90 genes were not reported up to now. Therefore, this study will identify the HSP90 gene family in the cucumber genome with bioinformatics methods, and analyze the physicochemical characteristics, chromosomal location, phylogenetic tree, gene structure, conserved motifs, homologous gene pairs, synteny, and *cis*-elements in the promoter of cucumber HSP90 genes. Furthermore, with the big data of cucumber transcriptome sequencing, the tissue-specific expression analysis of cucumber HSP90 family genes and their expression pattern analysis in response to the different stresses will be carried out. This study will preliminarily explore the relevant roles of cucumber HSP90 genes in response to abiotic and biotic stresses, which will create an important foundation for further research on the biological function of cucumber HSP90 genes, and provide a clear reference for cucumber resistance molecular breeding.

## Materials and Methods

### Genome-Wide Identification of HSP90 Genes in Cucurbitaceae Crops

The whole genome (dna, cds, pep, and gff3) of eight Cucurbitaceae crops including cucumber, watermelon, melon, *Cucurbita maxima*, bottle gourd, wax gourd, luffa, and bitter gourd was downloaded from the Cucurbit Genomic Database^1^ and CNSA database.^2^ The Hidden Markov Model (HMM) profile of the HSP90 domain (PF00183) was downloaded from the Pfam database.^3^ This model was used as a query to search for HSP90 proteins in the eight Cucurbitaceae crop protein files based on an expected value (E-value) cutoff of 1 × 10^−5^ in HMMER 3.0 (Finn et al., 2011). Subsequently, all the obtained HSP90 protein sequences were validated with the search results from the SMART^4^ and NCBI CDD^5^ databases. The protein sequences containing HSP90 and HATPase_c structure domains were finally determined as HSP90 proteins. The protein sequences of confirmed HSP90 family members were analyzed with the online tool ExPASy^6^ to predict their physicochemical characteristics. To obtain chromosomal distribution of cucumber HSP90 genes, the DNA sequence of each HSP90 gene was used in Blastn search of the cucumber genome in the Cucurbit Genomics Database. The cucumber HSP90 genes were then mapped onto chromosomes using the software TBtools (Chen et al., 2020).

### Structural Analysis and Phylogenetic Tree Construction of HSP90 Genes in Cucumber

The exon/intron structures of the HSP90 genes identified in the eight Cucurbitaceae crops were generated using the online tool Gene Structure Display Server^7^ (Hu et al., 2015). The conserved motifs in the HSP90 proteins identified in the eight Cucurbitaceae crops were analyzed with MEME server^8^ (Bailey et al., 2006) using the following parameters: maximum number of motifs, 10; minimum motif width, 6; and maximum motif width, 100. The conserved motifs were annotated with the Pfam database. Based on the results of HSP90 genes identified in *Arabidopsis* (Krishna and Gloor, 2001) and rice (Hu et al., 2009), seven *Arabidopsis* HSP90 proteins and nine rice HSP90 proteins were downloaded from the *Arabidopsis* Information Resource^9^ and the Rice Genome Annotation Project^10^, respectively. Multiple alignments of HSP90 protein sequences of cucumber, *Arabidopsis*, and rice were performed by Muscle in MEGA X (Kumar et al., 2018) with default parameters. Then, a phylogenetic tree was constructed on the basis of the alignment results using the maximum likelihood method with the following parameters: LG+G model, partial deletion, and 1,000 bootstrap tests. Another phylogenetic tree was constructed with the HSP90 proteins identified in the eight Cucurbitaceae crops using the maximum likelihood method.

### Synteny Analysis of HSP90 Genes in Cucumber, *Arabidopsis*, and Rice

To understand the syntenic relationships among the cucumber HSP90 genes, *Arabidopsis* HSP90 genes, and rice HSP90 genes, synteny analysis of HSP90 genes in cucumber, *Arabidopsis*, and rice was conducted. MCScanX software (Wang et al., 2012) was used to analyze the syntenic relationships of the HSP90 genes in cucumber, *Arabidopsis*, and rice. Circos software (Krzywinski et al., 2009) was used to visualize the syntenic relationships of the HSP90 genes in cucumber, *Arabidopsis*, and rice.

### *Cis*-Regulatory Element Prediction for Cucumber HSP90 Gene Promoters

The promoter sequence (1.5 kb DNA sequence upstream of the start codon) of each HSP90 gene was extracted from the cucumber genome. *Cis*-acting regulatory elements in the promoters of each cucumber HSP90 gene were then analyzed using the PlantCARE database^11^ (Lescot et al., 2002). The predicted *cis*-acting regulatory elements were classified according to their regulatory functions.

### Expression Profile Analyses of HSP90 Genes in Different Tissues of Cucumber

The RNA-seq data (PRJNA80169; Li et al., 2011) for cucumber gene expression in different tissues (root, stem, male flower, female flower, ovary, leaf, and tendril) were deposited into the Cucurbit Genomic Database. The expression profiles, as fragments per kilobase per million reads (RPKM), of all cucumber HSP90 genes were retrieved from PRJNA80169. An expression heatmap of cucumber HSP90 genes in different tissues was drawn using the TBtools software with Euclidean distances and the complete linkage method of hierarchical clustering.

### Expression Profile Analyses of Cucumber HSP90 Genes in Response to Heat Treatment

Cucumber seeds were soaked overnight at room temperature. The germinated seeds were sow in the nursery tray at the same time. Seedlings were grown in a growth room at 25°C and 14 h light/10 h dark cycle for 2 weeks. For heat stress, uniform-sized seedlings were transferred to a growth chamber that maintained the temperature at 45°C. The leaves were collected at 0 (control), 3, 6, and 12 h (three repeats, the first true leaf was taken for each repeat). The collected samples were used for qRT-PCR analysis. The primers used in qRT-PCR for the six cucumber HSP90 genes are shown in Supplementary Table S1.

### Expression Profile Analyses of Cucumber HSP90 Genes in Response to Abiotic and Biotic Stresses With Publicly Available Transcriptome Data

For abiotic and biotic treatments, RNA-seq data available in the Cucurbit Genomic Database under the series accession numbers of PRJNA376073 (GA treatment; Zhang et al., 2017), PRJNA285071 (downy mildew infection; Burkhardt and Day, 2016), PRJNA321023 (powdery mildew infection; Xu et al., 2017), and the GEO database under the series accession number of GSE117867 (temperature and photoperiod treatment; Zhang et al., 2018) were selected to analysis the biological functions of cucumber HSP90 genes. The expression profiles of cucumber HSP90 genes were retrieved from different RNA-seq data, respectively. The expression heatmaps of cucumber HSP90 genes under different stresses were drawn with the TBtools software.

## Results

### Genome-Wide Identification and Chromosomal Location of HSP90 Family Genes in Cucumber

Based on the published cucumber genome information (ChineseLong_V2), a total of six members of the HSP90 gene family were identified using bioinformatics methods. The length of the encoded HSP90 proteins ranged from 631 (Csa1G569290) to 817 (Csa2G368880) amino acids, with CDS sizes of 1896–2,454 bp, and a molecular weight of 72.31–93.59 kD. The aliphatic index varied from 79.61 (Csa6G111370) to 85.08 (Csa1G569270). The theoretical isoelectric point (pI) of the six HSP90 proteins ranged from 4.86 (Csa5G135340) to 5.47 (Csa6G111370), indicating that all the cucumber HSP90 proteins were acidic (pI < 7.0). The instability indexes of Csa5G135340 and Csa6G111370 were greater than 40, suggesting that Csa5G135340 and Csa6G111370 were stable proteins. While the instability indexes of other HSP90 proteins were less than 40, which suggested that the other four HSP90 proteins were unstable. The grand average of hydropathicity (GRAVY) values of all HSP90 proteins were negative, indicating that all the cucumber HSP90 proteins were hydrophilic (Table 1).

**Table 1:** The physiochemical characteristics of HSP90 genes identified in the 8 Cucurbitaceae crops.

Organism	Gene ID	CDS size/bp	Number of amino acids/aa	Molecular weight/kD	pI	Instability index	Aliphatic index	Grand average of hydropathicity
Cucumber	Csa1G569270	2,100	699	79.93	4.99	35.19	85.08	−0.56
	Csa1G569290	1896	631	72.31	5.01	38.69	83.90	−0.57
	Csa2G368880	2,454	817	93.59	4.89	35.25	79.95	−0.72
	Csa3G183950	2,112	703	80.93	4.97	38.33	83.20	−0.61
	Csa5G135340	2,352	783	88.64	4.86	43.92	82.21	−0.53
	Csa6G111370	2,376	791	89.52	5.47	43.51	79.61	−0.53
Melon	MELO3C027461.2	792	263	29.86	4.73	30.94	86.77	−0.56
	MELO3C015295.2	261	86	9.54	4.23	39.66	79.42	−0.69
	MELO3C011021.2	2,271	756	87.02	5.21	32.54	83.66	−0.68
	MELO3C006935.2	2,295	764	87.89	5.06	38.15	86.78	−0.51
	MELO3C005179.2	2,352	783	88.95	4.90	44.14	82.09	−0.55
	MELO3C025976.2	1758	585	66.48	6.21	40.74	80.32	−0.51
Watermelon	Cla97C01G007690	2,370	789	89.56	4.89	43.38	81.81	−0.54
	Cla97C02G035700	2,364	787	89.15	5.58	38.27	79.90	−0.55
	Cla97C03G067590	2,100	699	80.03	5.01	34.70	84.94	−0.57
	Cla97C03G067600	2,100	699	80.03	5.01	34.70	84.94	−0.57
	Cla97C03G067620	780	259	29.56	4.84	43.24	82.86	−0.61
	Cla97C03G067630	1,296	431	49.47	5.22	35.21	84.13	−0.57
	Cla97C08G157760	2,454	817	93.45	4.88	33.33	79.95	−0.72
*Cucurbita maxima*	CmaCh02G002220	4,380	1,459	165.95	6.08	46.40	84.74	−0.42
	CmaCh04G021830	2,379	792	89.73	4.89	43.17	81.62	−0.50
	CmaCh05G003150	2,451	816	93.19	4.94	33.20	80.31	−0.68
	CmaCh06G006950	2097	698	79.79	4.97	34.47	85.20	−0.56
	CmaCh11G011980	2,106	701	80.27	4.99	33.59	84.15	−0.58
	CmaCh12G004010	2,559	852	97.73	5.00	33.39	81.37	−0.67
	CmaCh15G008490	2,370	789	89.70	4.94	43.32	81.67	−0.54
	CmaCh16G000980	2,115	704	80.87	4.98	30.09	82.26	−0.62
	CmaCh16G009910	2,103	700	80.29	4.97	37.85	84.10	−0.60
	CmaCh20G006760	1,122	373	41.71	8.94	47.81	84.75	−0.30
Bottle gourd	Lsi06G000940	3,243	1,080	122.20	8.47	41.19	86.03	−0.51
	Lsi06G000960	2,100	699	79.99	5.01	35.48	84.94	−0.57
	Lsi08G013090	2,562	853	97.63	4.92	32.96	80.23	−0.70
	Lsi09G007770	2,445	814	92.72	4.84	41.52	81.45	−0.53
	Lsi10G000540	4,416	1,471	167.61	6.10	46.52	83.20	−0.49
Wax gourd	Bhi02M001715	1,326	441	51.11	5.11	40.66	89.09	−0.52
	Bhi02M001717	1,266	421	48.74	5.10	39.67	88.93	−0.52
	Bhi04M000114	2,451	816	93.40	4.97	33.20	79.69	−0.72
	Bhi05M001813	2,112	703	80.92	5.01	36.89	82.65	−0.62
	Bhi10M001028	2,349	782	88.52	5.58	35.41	81.29	−0.52
	Bhi12M000916	2,352	783	88.85	4.95	43.46	82.55	−0.54
Luffa	Lcy01g016200	2,457	818	93.38	4.94	33.15	79.38	−0.71
	Lcy03g001820	2,100	699	79.97	5.00	32.79	84.81	−0.57
	Lcy03g001830	2,100	699	79.95	5.00	33.42	84.81	−0.56
	Lcy07g011100	2,388	795	90.11	5.52	36.90	80.82	−0.54
	Lcy09g008390	2,379	792	89.80	4.93	43.01	81.50	−0.54
	Lcy11g001270	2,112	703	80.87	4.99	38.20	81.82	−0.63
Bitter gourd	MC02g0401	2,199	732	83.24	5.30	41.58	80.85	−0.54
	MC04g1065	2,379	792	89.77	4.88	44.12	80.63	−0.56
	MC08g0753	2,454	817	93.21	4.89	30.40	82.01	−0.69
	MC10g0106	2,118	705	81.02	4.95	39.92	82.70	−0.62
	MC00g0429	2,106	701	80.10	4.96	34.64	83.87	−0.58
	MC00g0432	2,193	730	83.50	5.03	33.59	87.62	−0.48

Moreover, the HSP90 gene family in the other seven Cucurbitaceae crops including watermelon, melon, *Cucurbita maxima*, bottle gourd, wax gourd, luffa, and bitter gourd were also identified. Most Cucurbitaceae crops (diploid) had similar HSP90 members (5–7), which were similar to the HSP90 gene members in cucumber (6; Table 1). Whereas *Cucurbita maxima* was tetraploid, which had 10 HSP90 gene members. From the numbers of HSP90 gene family in different Cucurbitaceae crops, the HSP90 gene family in Cucurbitaceae crops was conservative.

Based on the chromosomal positions of the HSP90 genes annotated in the cucumber genome, the chromosomal locations of six HSP90 genes were marked on the physical maps of cucumber. In the cucumber genome, the six HSP90 family genes were distributed on five chromosomes of cucumber. Two HSP90 genes were located on chromosome 1, which contained the most HSP90 genes, whereas chromosomes 2, 3, 5, and 6 each contained only one HSP90 gene (Supplementary Figure S1).

### Phylogenetic, Gene Structure, and Conserved Motifs Analysis of Cucumber HSP90 Proteins

Based on the unrooted phylogenetic tree of the six HSP90 protein sequences, the cucumber HSP90 gene family was classified into two major subgroups, namely subgroup A clustered with Csa1G569270, Csa1G569290, and Csa3G183950 and subgroup B clustered with Csa2G368880, Csa5G135340, and Csa6G111370 (Supplementary Figure S2). The exon/intron distribution showed that the number of exons in the six cucumber HSP90 genes varied from 1 (Csa1G569270) to 20 (Csa6G111370). The average number of exons in the HSP90 genes in subgroup A was the lowest, only 3.7, while the average number of exons in the HSP90 genes in subgroup B was the highest, 18 (Supplementary Figure S2). It is noteworthy that closely related HSP90 genes in the phylogenetic tree showed similar distribution of exon/intron, suggesting that the functions of HSP90 genes in the same subgroup were similar. A total of 10 conserved motifs were identified in all six cucumber HSP90 proteins using the MEME web server. Based on the annotation information on the Pfam database, motif 6 was annotated as HATPase_c, motif 4 had no annotation, and the other eight motifs were all annotated as HSP90 (Supplementary Table S2). In subgroup A, in addition to 10 motifs, each HSP90 protein contained one more motif 8 at the 3′ end, and the order of all motifs was consistent. In subgroup B, each HSP90 protein contained 10 motifs, and the order of all motifs was consistent (Supplementary Figure S2). These results revealed that the type, order, and number of motifs were similar in the HSP90 proteins within the same subgroup, indicating functional similarities among members of the same subgroup. Whereas the differences in motif distribution in HSP90 proteins among different subgroups revealed that the functions of these genes might have diverged during evolution.

### Evolutionary Relationships and Classification of Cucumber HSP90 Family Genes

**Figure 1:**
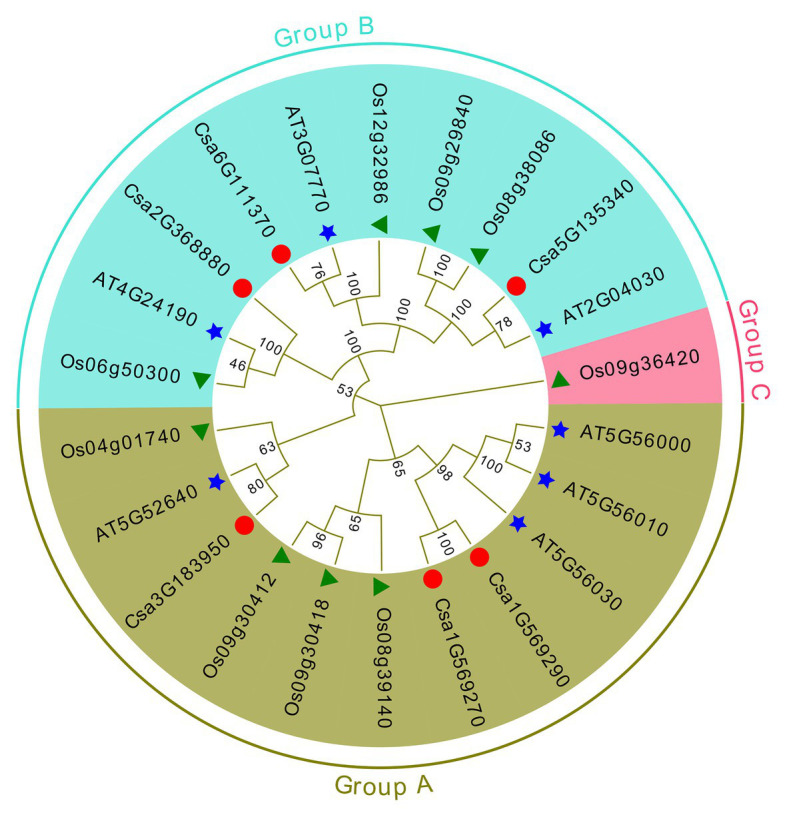
Phylogenetic analysis of HSP90 proteins from cucumber, *Arabidopsis*, and rice.

To examine the evolutionary relationships and classification of cucumber HSP90 genes, a maximum likelihood phylogenetic tree was generated based on the multiple sequence alignment of 22 full-length HSP90 protein sequences from cucumber (6), *Arabidopsis* (7), and rice (9). The evolutionary tree was divided into three subgroups: A, B, and C. There were, respectively, three cucumber HSP90 genes in the A and B subgroups (Figure 1), which were consistent with the results of the cluster analysis of HSP90 family genes in the cucumber (Supplementary Figure S2). Whereas subgroup C only contained one rice HSP90 gene (Figure 1), indicating that this gene was significantly different from the HSP90 genes in cucumber and *Arabidopsis*. Homologous genes were often clustered, indicating that cucumber HSP90 genes have a closer evolutionary relationship to those of *Arabidopsis* than to those of rice. The HSP90 genes with a evolutionary relationship were similar in gene structure and function, which suggested that the biological function of cucumber HSP90 genes could be predicted based on similar genes in *Arabidopsis*.

**Figure 2:**
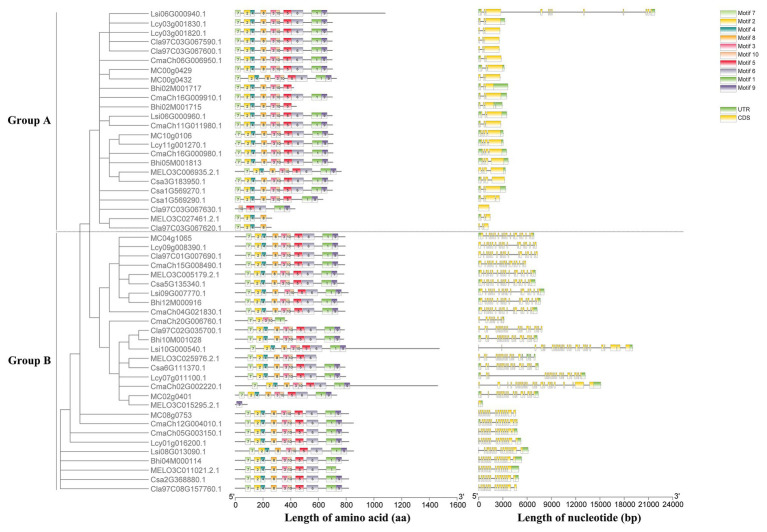
Phylogenetic tree, conserved motifs of HSP90 proteins, and exon/intron structures of HSP90 genes in the eight Cucurbitaceae crops.

A maximum likelihood phylogenetic tree was also generated based on the multiple sequence alignment of 52 full-length HSP90 protein sequences from cucumber and the other seven Cucurbitaceae crops (Figure 2). The phylogenetic tree showed that the cucumber HSP90 proteins were closely clustered with melon HSP90 proteins. The subgroup A HSP90 proteins were functionally conservative in different Cucurbitaceae crops, which contained 1–2 subgroup A HSP90 proteins in each Cucurbitaceae crops. The subgroup B HSP90 proteins were functionally differentiated in different Cucurbitaceae crops. The HSP90 proteins in the same subgroup showed similar conserved motifs and similar distribution of exon/intron.

### Homologous Gene Pairs and Synteny Analysis of HSP90 Family Genes

**Figure 3:**
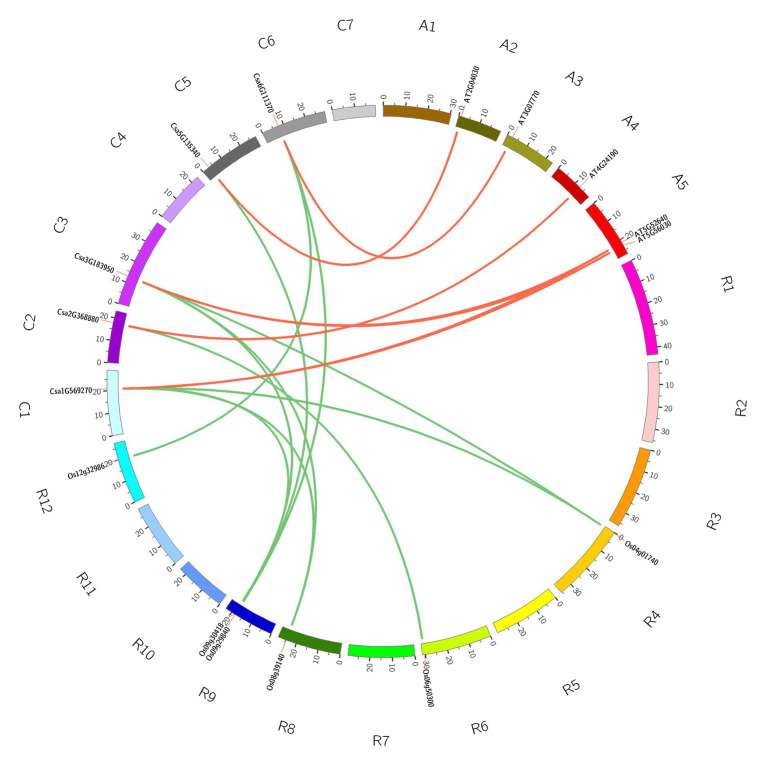
Syntenic relationships of HSP90 gene family in cucumber, *Arabidopsis*, and rice.

Tandem and segmental duplications are reported to be the two main mechanisms underlying gene family expansion (Cannon et al., 2004). The analysis of cucumber HSP90 gene duplication events showed that there was only one pair of paralogous genes, namely one pair of tandem duplication genes (Csa1G569270/Csa1G569290) in the cucumber HSP90 gene family, and segmental duplications were not found. Orthologous gene pairs can provide effective information about evolutionary relationships between species (Wei et al., 2015). We therefore investigated the orthologous genes of the HSP90 family between cucumber and *Arabidopsis*, and cucumber and rice with synteny analysis. The results revealed that five cucumber HSP90 genes and five *Arabidopsis* HSP90 genes were orthologous genes with seven syntenic relationships. Five cucumber HSP90 genes and six rice HSP90 genes were orthologous genes with 10 syntenic relationships (Figure 3). Only one cucumber HSP90 gene Csa1G569290 did not form the syntenic relationships with neither *A. thaliana* nor rice, which suggested that Csa1G569290 was conservative in the cucumber HSP90 gene family.

### Analysis of the Promoter Sequences of Cucumber HSP90 Genes

**Figure 4:**
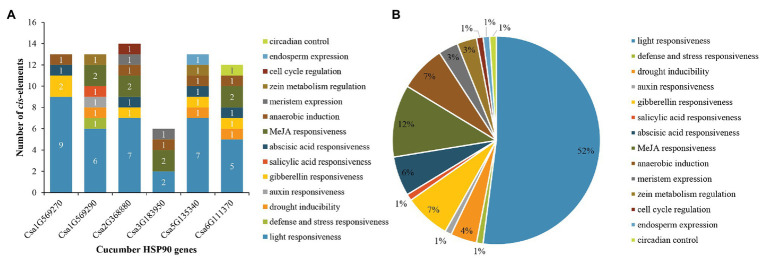
Distribution of *cis*-elements in the promoters of cucumber HSP90 genes. **(A)** The number of various *cis*-elements in the promoters of each cucumber HSP90 gene. **(B)** The relative proportions of different *cis*-elements in the promoters of cucumber HSP90 genes are indicated by the pie chart. *Cis*-elements sharing identical or similar functions are represented by the same color.

For cucumber HSP90 genes, 14 main types of *cis*-elements were identified in their promoter sequences. The number of various *cis*-elements in the promoters of each cucumber HSP90 gene is shown in Figure 4A. The largest number of *cis*-elements observed across the six HSP90 genes was associated with light-responsiveness, such as ACE, G-box, and MRE. Most notably, light-responsive *cis*-elements constituted more than half (up to 52%) of the presumptive *cis*-elements (Figure 4B). In addition, various *cis*-elements involved in hormone response (e.g., MeJA, gibberellins, abscisic acid, auxin, and salicylic acid), stress response (e.g., drought, defense, and stress), anaerobic induction, meristem expression, zein metabolism regulation, cell cycle regulation, endosperm expression, and circadian control were also identified in the promoter sequences of cucumber HSP90 genes. The different members of *cis*-elements were present in the promoter regions of different HSP90 genes, indicating that cucumber HSP90 genes performed multiple functions in the process of plant growth and development.

### Tissue-Specific Expression Profile Analyses of Cucumber HSP90 Genes

**Figure 5:**
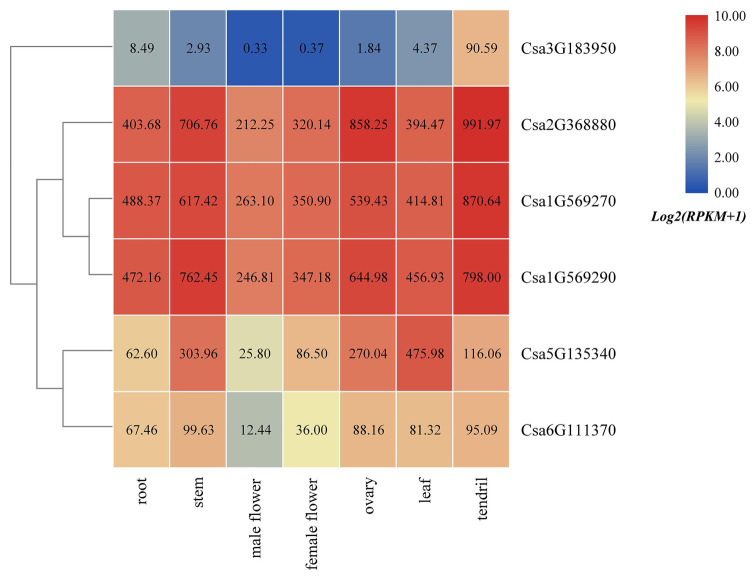
The expression heatmap of the HSP90 gene family in different tissues of cucumber. The data in the boxes indicate original RPKM values.

Based on the published transcriptome sequencing data from different tissues of cucumber (PRJNA80169; Li et al., 2011), the expression heatmap of HSP90 family genes in different tissues of cucumber was drawn (Figure 5). The results revealed that the HSP90 gene Csa3G183950 was only highly expressed in tendril, but was low or not expressed in other tissues. Three HSP90 genes, Csa1G569270, Csa1G569290, and Csa2G368880, were highly expressed in root, stem, male flower, female flower, ovary, leaf, and tendril, indicating that these three HSP90 genes played an important role in cucumber growth and development. The expression levels of HSP90 genes Csa5G135340 and Csa6G111370 were relatively low in male flower, but were high in other tissues.

### Expression Profile Analyses of Cucumber HSP90 Genes Under Heat Stress

**Figure 6:**
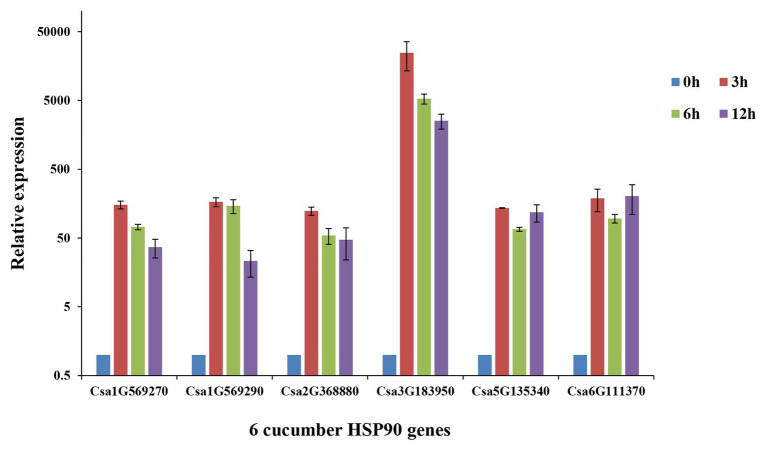
Relative expression levels of six cucumber HSP90 gene after 0, 3, 6, and 12 h treatment of heat stress in cucumber seedlings by qRT-PCR analysis. Data are displayed using the *CsActin* gene as an internal control with three biological and three technical replicates. Values are the mean ± SD.

The qRT-PCR analysis of the six cucumber HSP90 genes showed that all of the six cucumber HSP90 genes responded to heat stress (Figure 6). Among them, Csa1G569270, Csa1G569290, Csa2G368880, and Csa3G1839050 were highly expressed after 3 h of heat stress, and then downregulated along with the time. The two cucumber HSP90 genes Csa5G13540 and Csa6G111370 were highly expressed after 3 h of heat stress, downregulated at 6 h, and then continued to upregulate at 12 h of heat stress. After the 12 h treatment of heat stress, the expression levels of the six cucumber HSP90 genes were still higher than that of blank control. The results indicated that all the cucumber HSP90 genes quickly responded to heat stress with a long response time.

### Expression Profile Analyses of Cucumber HSP90 Genes Under Temperature and Photoperiod Treatment

**Figure 7:**
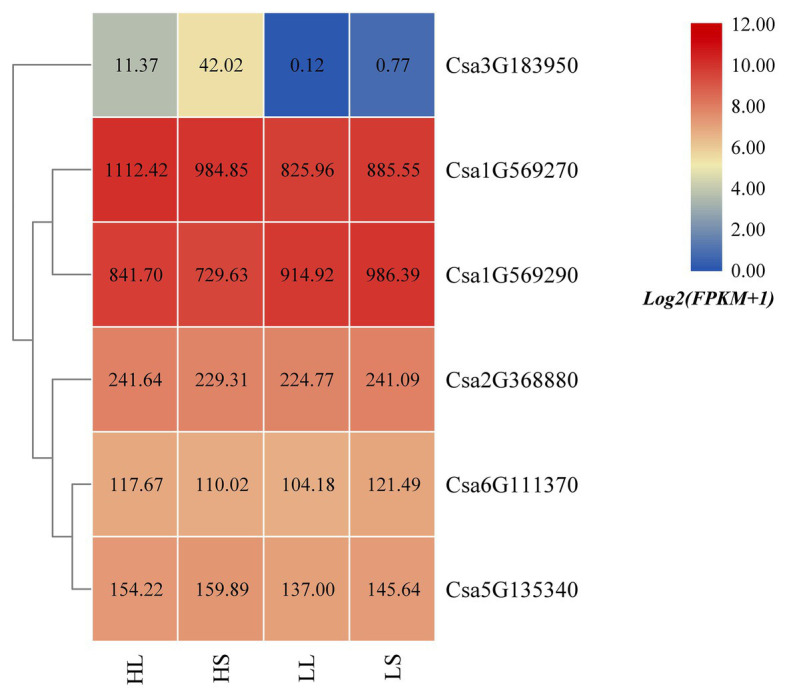
The expression heatmap of the cucumber HSP90 gene family under temperature and photoperiod treatment. HL is high temperature and long day; HS is high temperature and short day; LL is low temperature and long day; LS is low temperature and short day. The data in the boxes indicate original FPKM values.

Based on the published cucumber transcriptome sequencing data under temperature and photoperiod treatment (GSE117867; Zhang et al., 2018), the expression heatmap of the cucumber HSP90 gene family under temperature and photoperiod treatment was drawn (Figure 7). It was found that the expression levels of Csa1G569270 and Csa3G183950 at high temperature were higher than that at low temperature, indicating that these two HSP90 genes responded to high temperature stress. The expression level of the Csa1G569290 gene at high temperature was lower than that at low temperature, suggesting that the HSP90 gene Csa1G569290 responded to low temperature stress. The expression levels of Csa1G569270 and Csa1G569290 genes under high temperature and long day treatment were higher than those under high temperature and short day treatment, and the expression levels of these two HSP90 genes under low temperature and long day treatment were lower than those under low temperature and short day treatment, which indicated that these two HSP90 genes were associated with photoperiod response. The expression levels of Csa2G368880, Csa6G111370, and Csa5G135340 under temperature and photoperiodic stresses were not changed, indicating that these three HSP90 genes did not respond to temperature and photoperiodic stresses.

### Expression Profile Analyses of Cucumber HSP90 Genes Under GA Treatment

**Figure 8:**
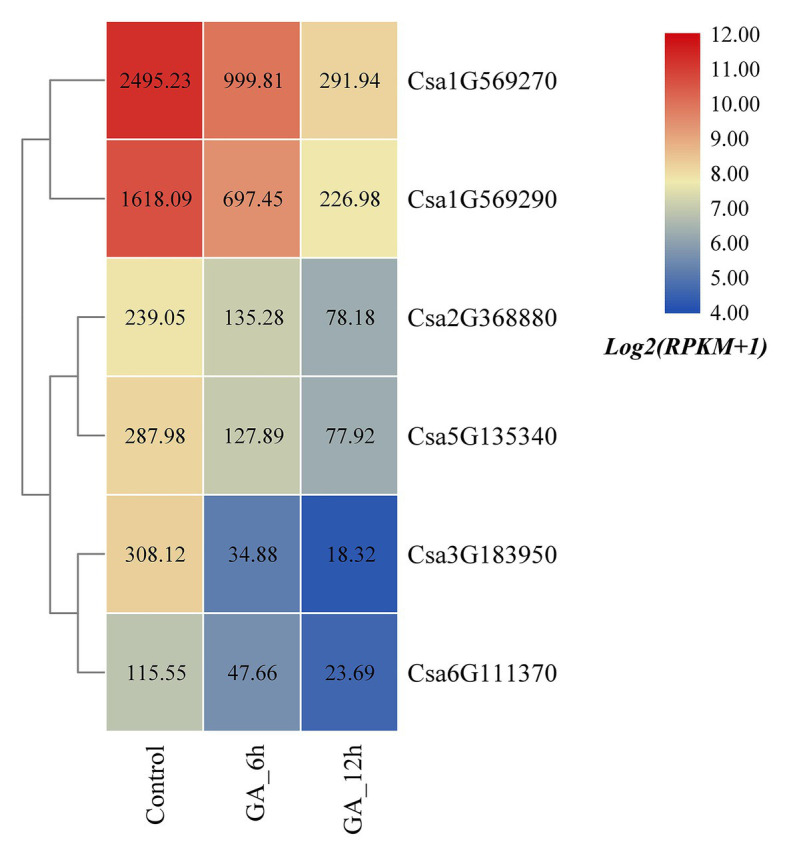
The expression heatmap of the cucumber HSP90 gene family under GA treatment. Control is the control sample; GA_6h is the sample treated with GA for 6 h; GA_12h is the sample treated with GA for 12 h. The data in the boxes indicate original RPKM values.

Based on the published transcriptome sequencing data of cucumber treated with gibberellin (GA) at different times (PRJNA376073; Zhang et al., 2017), the expression heatmap of the cucumber HSP90 gene family under GA treatment was plotted (Figure 8). The results revealed that the expression levels of all the six cucumber HSP90 family genes were significantly downregulated under GA treatment when compared with the control materials. With the extension of GA treatment time, the expression levels of the six HSP90 genes were all gradually decreased, indicating that these six cucumber HSP90 family genes were closely related to GA signal transduction.

### Expression Profile Analyses of Cucumber HSP90 Genes in Response to Downy Mildew Infection

**Figure 9:**
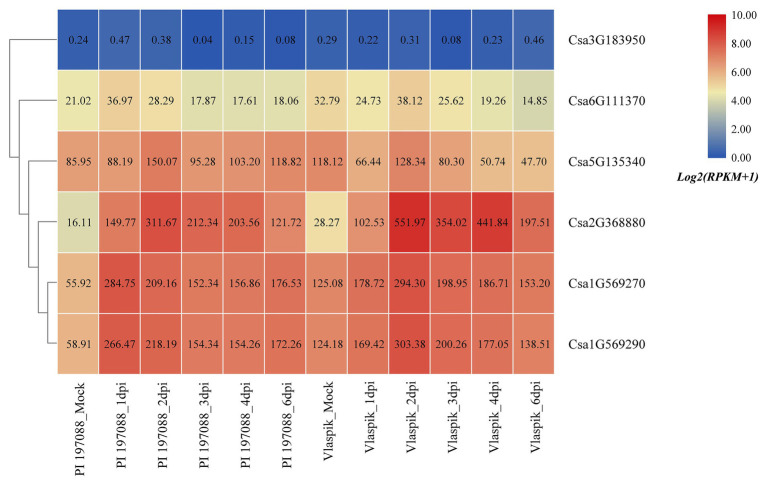
The expression heatmap of the cucumber HSP90 gene family under the treatment of downy mildew inoculation. PI 197088 is the downy mildew resistant cucumber plant; Vlaspik is the downy mildew susceptible cucumber plant. Mock is the control sample. dpi means the days post inoculation. The data in the boxes indicate original RPKM values.

Based on the published transcriptome sequencing data of resistant and susceptible cucumber plants inoculated with downy mildew for different time points (PRJNA285071; Burkhardt and Day, 2016), the expression heatmap of cucumber HSP90 genes under the treatment of downy mildew inoculation was drawn (Figure 9). The results showed that the Csa3G183950 gene was not expressed in resistant and susceptible cucumber materials, which may be related to the low or nil expression level of the Csa3G183950 gene in the leaf tissue. The expression levels of the Csa6G111370 gene in resistant and susceptible cucumber plants were not changed significantly after inoculation with the downy mildew pathogen, indicating that the Csa6G111370 gene did not function in response to downy mildew inoculation. The expression level of the Csa5G135340 gene was slightly upregulated in resistant cucumber material after inoculation with downy mildew, but was significantly downregulated in susceptible cucumber material after inoculation with downy mildew, which suggested that the expression patterns of the Csa5G135340 gene were different in the resistant and susceptible cucumber plants after inoculation with downy mildew. After inoculation with downy mildew, the expression levels of the Csa2G368880 gene in resistant and susceptible cucumber plants were all upregulated at first, and then decreased slowly, but the expression level on the 6th day was still seven times higher than that of the mock plant. After inoculation with downy mildew, the expression levels of the Csa1G569270 and Csa1G569290 genes in resistant and susceptible cucumber plants were upregulated first, and then decreased slowly. Six days post inoculation with downy mildew, the expression levels of the Csa1G569270 and Csa1G569290 genes in resistant cucumber plants were still about three times the expression level in the control plant, whereas the expression levels of these two genes in susceptible cucumber plants were almost consistent with the expression levels in the control plant, which indicated that these two HSP90 genes may be involved in the regulation pathways related to cucumber downy mildew resistance.

### Expression Profile Analyses of Cucumber HSP90 Genes in Response to Powdery Mildew Infection

**Figure 10:**
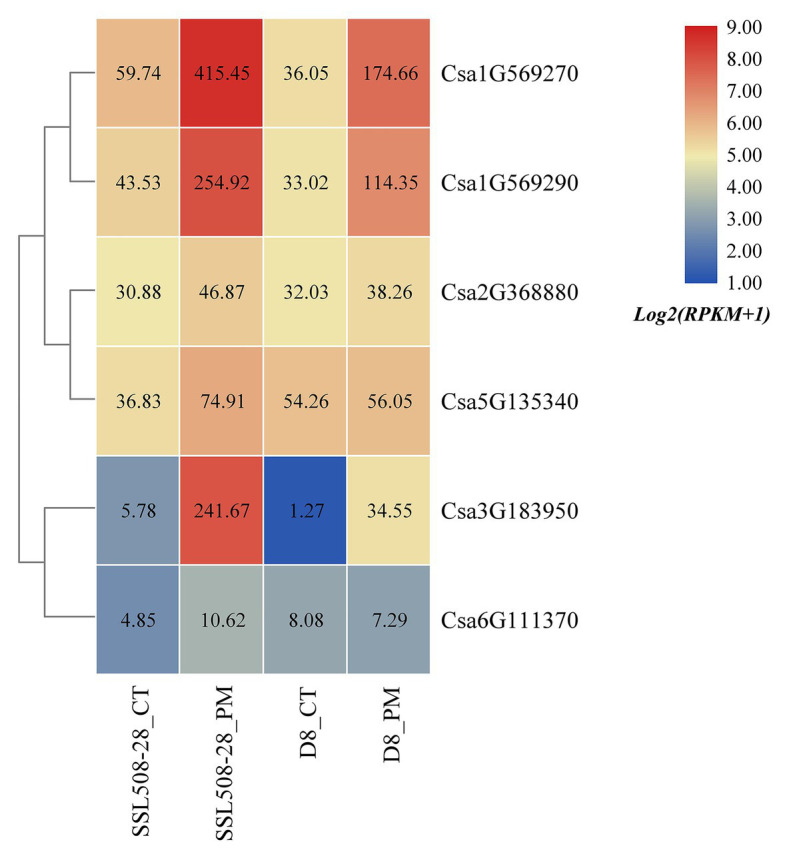
The expression heatmap of the cucumber HSP90 gene family under the treatment of powdery mildew inoculation. SSL508-28 is the powdery mildew resistant cucumber plant; D8 is the powdery mildew susceptible cucumber plant. CT is the control sample. PM means powdery mildew inoculation. The data in the boxes indicate original RPKM values.

Based on the published transcriptome sequencing data of resistant and susceptible cucumber plants inoculated with powdery mildew for different time points (PRJNA321023; Xu et al., 2017), the expression heatmap of cucumber HSP90 genes under the treatment of powdery mildew inoculation was drawn (Figure 10). The results showed that the expression levels of the Csa1G569270, Csa1G569290, and Csa3G183950 genes in resistant and susceptible cucumber plants were all significantly upregulated after inoculation with powdery mildew, and the differential expression ratio in the resistant cucumber was significantly higher than that in the susceptible cucumber, indicating that these three HSP90 genes may be associated with cucumber powdery mildew resistance. After inoculation with powdery mildew, the expression level of Csa5G135340 doubled in the resistant cucumber plant, but was not changed in the susceptible cucumber plant, suggesting that this HSP90 gene may also be involved in the regulation pathway related to cucumber powdery mildew resistance. The expression level of the Csa2G368880 gene in resistant and susceptible cucumber plants was not changed after inoculation with powdery mildew. The expression levels of the Csa6G111370 gene in resistant and susceptible cucumber plants were low, and the difference was not significant. These results revealed that these two HSP90 genes Csa2G368880 and Csa6G111370 did not function in response to powdery mildew infection in cucumber.

## Discussion

HSP90 is a class of chaperone protein that is highly conserved in prokaryotes and all eukaryotes, which is widely involved in signal transduction, cell cycle, and other important biological processes in organisms (Pearl and Prodromou, 2006). To date, the function of HSP90 genes have been widely investigated in animal and fungus systems, but lagged behind in plants. Recently, with the continuous development and extensive use of genome sequencing technology, more and more plant genome information has been published (Kersey, 2019). Many important gene families have been identified in plants. The HSP90 gene family has been successively identified in the model plants *Arabidopsis* (Krishna and Gloor, 2001) and rice (Hu et al., 2009). Subsequently, the HSP90 gene family was also reported in some important vegetable crops, such as tomato (Liu et al., 2014) and pepper (Wang et al., 2020). Cucumber is an important vegetable crop widely cultivated in the world, whose cultivated area is next only to tomato and onion [UN Food and Agriculture Organization Corporate Statistical Database (FAOSTAT), 2017]. The cucumber genome was sequenced as early as 2009, which included high-quality genomic information (Huang et al., 2009). However, the genome-wide identification of the HSP90 gene family and their expression patterns analysis has not been conducted in cucumber. This greatly limited the study of the biological functions of HSP90 genes in cucumber. Therefore, in this study, with the cucumber genomic information and transcriptomic sequencing big-data, the HSP90 family genes were identified in cucumber, and the tissue-specific expression patterns of the cucumber HSP90 genes and expression profiles of the cucumber HSP90 genes in response to different stresses were analyzed. The results will provide vital references for further study of the biological functions of cucumber HSP90 genes and provide a theoretical basis for cucumber resistance molecular breeding.

In this study, a total of six HSP90 family genes were identified in cucumber, which was less than the members of the HSP90 gene family identified in *Arabidopsis thaliana* (7; Krishna and Gloor, 2001), rice (9; Hu et al., 2009), poplar (10; Zhang et al., 2013), tomato (7; Liu et al., 2014), and pepper (7; Wang et al., 2020). This may be one of the reasons for the differences in heat resistance among different plants. We also identified the HSP90 gene family in the other seven Cucurbitaceae crops including watermelon, melon, *Cucurbita maxima*, bottle gourd, wax gourd, luffa, and bitter gourd. Most Cucurbitaceae crops (diploid) had similar HSP90 members (5–7) with cucumber, except for the tetraploid crop *Cucurbita maxima* (10). It indicated that the HSP90 gene family in Cucurbitaceae crops was conservative. However, the number of cucumber HSP90 genes identified in the report of Li et al. (2020) was only four, which may be due to the different criterion for identification for the HSP90 gene family. Physicochemical characteristics analysis showed that all six HSP90 proteins in cucumber were acidic. This finding was consistent with the HSP90 proteins in other plants, such as *Arabidopsis*, rice, *Populus*, and tomato. While, except for one HSP90 protein, most of the HSP90 proteins in pepper were acidic. This indicated that the HSP90 proteins were conserved in different plant species. Phylogenetic tree analysis of 22 HSP90 genes derived from *Arabidopsis*, rice, and cucumber were divided into three subgroups: A, B, and C. The six HSP90 genes were classified into A and B subgroups, which was similar to the results of phylogenetic analysis for HSP90 genes in *Arabidopsis* (Krishna and Gloor, 2001), rice (Zhang et al., 2016), and *Populus* (Hu et al., 2009). According to the phylogenetic and gene structure analysis, most of the HSP90 genes in each subgroup showed a similar exon/intron structure and conserved motif, the gene structure of HSP90 genes between subgroups A and B were significantly different, which indicated that the evolution might not only affect the gene function, but also the gene structure (Babenko et al., 2004; Roy and Penny, 2007). The similar gene structure in the same subgroup and the different gene structure between different subgroups were also identified in the HSP90 gene family of other plants, such as pepper and *Populus*. Only one rice HSP90 gene was classified into the C subgroup, which was consistent with the results of phylogenetic analysis of rice HSP90 genes (Zhang et al., 2016).

According to the synteny analysis of HSP90 family genes in *Arabidopsis*, rice, and cucumber, only one pair of paralogous genes in the cucumber HSP90 gene family, namely one pair of tandem duplication genes (Csa1G569270/Csa1G569290), was detected, and segmental duplications were not found, indicating that the expansion of the HSP90 gene in cucumber was mainly caused by tandem duplication genes. Only one cucumber HSP90 gene, Csa1G569290, was conservative and did not have collinearity with the HSP90 genes in *Arabidopsis* and rice. Whereas the other five cucumber HSP90 genes showed a variety of collinearity with the five HSP90 genes in *Arabidopsis* and the six HSP90 genes in rice, which were orthologous genes. These results indicated that the expansion of the HSP90 gene family in each species was performed in a specific way. This phenomenon was also common in the studies of other plant gene families (Zhang et al., 2005; Jain et al., 2006). The promoter analysis showed that the promoter of cucumber HSP90 genes contained various regulation elements, such as *cis*-acting regulatory elements essential for hormone response, stress response, meristem expression, cell cycle regulation, and so on, which further suggested that HSP90 genes play key roles in signal transduction, cell-cycle control, protein degradation, genomic silencing, and protein trafficking (Richter and Buchner, 2001; Young et al., 2001).

In recent years, the rapid development of high-throughput sequencing technologies has dramatically increased the throughput of sequence generation and decreased the overall cost (Wang et al., 2010). Many researchers have conducted a large number of cucumber transcriptome sequencing studies, forming the big data of cucumber transcriptome sequencing. The big data of cucumber transcriptome sequencing have been validated with the qRT-PCR analysis and peer-reviewed, which could be considered as reliable data. Therefore, the effective utilization of these big data of cucumber transcriptome sequencing can not only reduce the research cost, but also facilitate the deep mining of the data of each transcriptome sequence. Besides, integrated analysis of the big data of cucumber transcriptome sequencing under different treatments is beneficial for studying the biological functions of cucumber genes. In this study, the published cucumber transcriptome sequencing big data were used to analyze the tissue-specific expression of six identified cucumber HSP90 family genes and their expression patterns in response to different stresses. The tissue-specific expression analysis showed that only cucumber HSP90 gene Csa3G183950 was highly expressed in tendril and low or not expressed in the other tissues, while the other five HSP90 genes were expressed or even highly expressed in all tissues. These results indicated that the functions of cucumber HSP90 genes were diverse in different tissues. Through the expression profile analyses of cucumber HSP90 family genes in response to abiotic stresses, two HSP90 genes, Csa1G569270 and Csa3G183950, were identified to respond to high temperature stress, which was consistent with the functions of *Arabidopsis* homologous HSP90 genes AT5G56010 and AT5G52640 (Sangster et al., 2007). The expression profiles of Csa1G569270 and Csa3G183950 genes under heat stress were also validated by the qRT-PCR analysis. While the expression levels of some other cucumber HSP90 genes were not changed in the publicly available transcriptome data, but upregulated in the qRT-PCR analysis. This may be due to the complex treatments in the transcriptome data which combined temperature and photoperiod. Cucumber HSP90 gene Csa1G569290 responded to low temperature stress, which showed similar functions with *Arabidopsis* HSP90 genes HSP90.2 (AT5G56010) and HSP90.3 (AT5G56030; Bao et al., 2014). Two cucumber HSP90 genes, Csa1G569270 and Csa1G569290, were related to photoperiod regulation. It had also been reported that *Arabidopsis* HSP90 genes were involved in photoperiod regulation (Ma, 2014). All the cucumber HSP90 genes were downregulated after GA treatment. This finding has not been reported in the other plant HSP90 genes. However, it has been reported that jasmonic acid (JA) signal was involved in the formation of the SGT1b-HSP70-HSP90 chaperone complex, and the SGT1b protein is necessary for plant response to JA, IAA, and GA stresses (Zhang et al., 2015). In addition, we found that the promoter sequence of cucumber HSP90 genes contained GA response elements. Therefore, it is believed that HSP90 genes were also involved in response to GA stress. Through the expression patterns analysis of cucumber HSP90 family genes in response to biotic stresses, two cucumber HSP90 genes, Csa1G569270 and Csa1G569290, were involved in the regulatory pathway related to cucumber downy mildew resistance. Zhang et al. (2019) reported that seven heat shock proteins (not clear whether they were HSP90) were differentially accumulated in cucumber under *Pseudoperonospora cubensis* infection. Previous studies had also found that wheat HSP90 genes were associated with disease resistance (Wang et al., 2011). Four cucumber HSP90 genes, Csa1G569270, Csa1G569290, Csa3G183950, and Csa5G135340, were involved in powdery mildew resistance in cucumber. It was also found that HSP90 genes performed the function of powdery mildew resistance in barley (Hein et al., 2005). Overall, it was found that two cucumber HSP90 genes, Csa1G569270 and Csa1G569290, were differentially expressed in response to abiotic stresses and biotic stresses, indicating that these two genes played an important role in cucumber growth and development.

## Conclusion

In this study, HSP90 family genes were identified and characterized in the cucumber for the first time. A total of six HSP90 genes in cucumber were identified and characterized by the systematic analyses of physicochemical characteristics, chromosomal location, gene structure, conserved motif, phylogenetic tree, homologous gene pairs, synteny, and *cis*-elements in the promoters, which showed a clear evolutionary history for this family in cucumber. The expression patterns of cucumber HSP90 genes in different tissues and stresses responses were different, which coordinately regulated the growth and development of cucumber. Two cucumber HSP90 genes, Csa1G569270 and Csa1G569290, were both differentially expressed in response to abiotic and biotic stresses, which means these two HSP90 genes play important roles in the process of cucumber growth and development. This study could not only provide a scientific foundation for the comprehensive understanding of the cucumber HSP90 gene family, but could also be helpful in screening candidate genes for breeding new cucumber varieties with a high yield and stresses resistance.

## Data Availability Statement

The original contributions presented in the study are included in the article/Supplementary Material, further inquiries can be directed to the corresponding author.

## Author Contributions

LJ conceived and designed the project. KZ, SH, and YS conducted the bioinformatics analysis. KZ, QG, and SJ performed the analysis of cucumber transcriptome sequencing big-data. KZ and XL wrote the paper. All authors reviewed and approved the final manuscript.

### Conflict of Interest

The authors declare that the research was conducted in the absence of any commercial or financial relationships that could be construed as a potential conflict of interest.

## Footnotes

^1^http://www.icugi.org/

^2^https://db.cngb.org/cnsa/home/

^3^http://pfam.xfam.org/family/PF00183/hmm

^4^http://smart.embl.de/smart/batch.pl

^5^https://www.ncbi.nlm.nih.gov/Structure/cdd/wrpsb.cgi

^6^http://web.expasy.org/protparam/

^7^http://gsds.cbi.pku.edu.cn/

^8^http://memesuite.org/

^9^http://www.arabidopsis.org/index.jsp

^10^http://rice.plantbiology.msu.edu/cgi-bin/ORF_infopage.cgi

^11^http://bioinformatics.psb.ugent.be/webtools/plantcare/html/
