# Supplementary material for: Genome-Wide Characterization of HSP90 Gene Family in Cucumber and Their Potential Roles in Response to Abiotic and Biotic Stresses
Source: Front Genet. 2021 Feb 4;12:584886. doi: 10.3389/fgene.2021.584886 (PMC7889589; doi:10.3389/fgene.2021.584886)
Supplement: Supplementary file 1 [file Table_1.docx]

***Supplementary Material***

**1 Supplementary Figures**


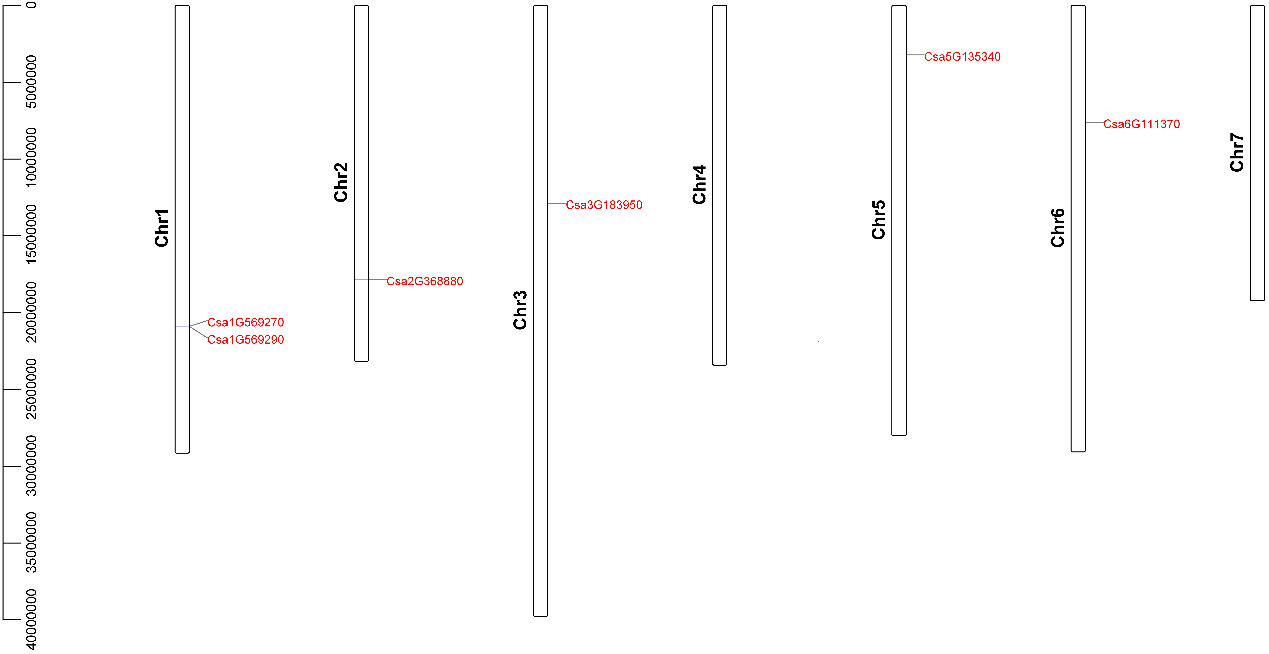


**Supplementary Figure 1.** The chromosomal distribution of cucumber HSP90 genes.

**
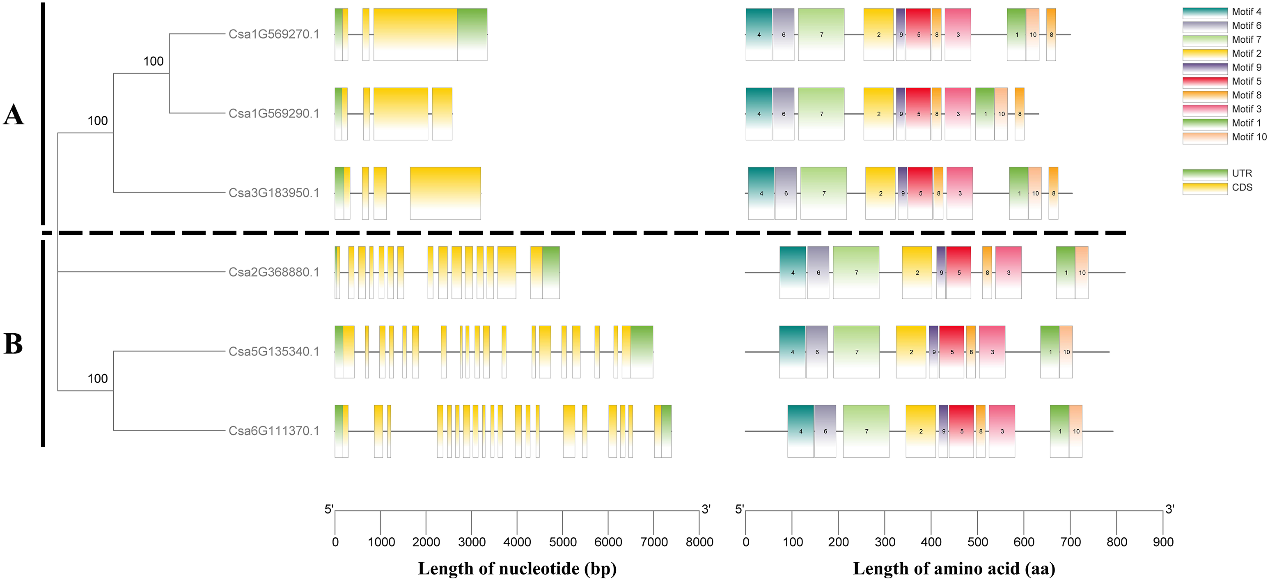
**

**Supplementary Figure 2.** Phylogenetic tree, exon/intron structures of HSP90 genes and a schematic diagram of the conserved motifs of HSP90 proteins in cucumber.

**2 Supplementary Tables**

**Supplementary Table 1.** List of primers used in quantitative RT-PCR for the 6 cucumber HSP90 genes.

| **Gene ID** | **Forward primer 5' to 3'** | **Reverse primer 5' to 3'** |
| --- | --- | --- |
| *Csa1G569270* | ACGAGTGGTCGTTGGTGAAC | AGAGAAGTGCTTTACGGCCA |
| *Csa1G569290* | CAAGAAGAAGGTTGAGAAGGTTG | CATGCTGTTGTCCTTCAATGCT |
| *Csa2G368880* | GTCCCAAAGAGGTCACGGAG | GGAGGCACAAACAGAACAGC |
| *Csa3G183950* | CTCTCCTTGCTGCTTGGTCA | TGTAAGCGCCCATGCTGTTA |
| *Csa5G135340* | CGGTGGTACTCGCTTGAGTT | CGCCTCACACCTAACAGCAA |
| *Csa6G111370* | TTCGCTCTGGTGGAGCTTAC | CACCGAATGGGAGAGGTGAG |

**Supplementary Table 2.** The motifs information of cucumber HSP90 proteins.

| **Motif** | **Sequence** | **Number of amino acid** | **Pfam annotation** |
| --- | --- | --- | --- |
| motif 1 | DSPCVLVTGEYGWSANMERIMKAQALKDNSMAGYMRSKKTM | 41 | HSP90 |
| motif 2 | HEWELVNKQKPIWMRKPEEITKEEYAAFYKSLTNDWEEHLAVKHFSVEGQLEFKAILFVPKRAPF | 65 | HSP90 |
| motif 3 | AELLRFYSTKSGDELTSLDDYVTRMKEGQKDIYYITGESKKAVENSPFLEKLKKKGY | 57 | HSP90 |
| motif 4 | ADTETFEFQAEINRLLDLIINSLYSNKEIFLRELISNASDALDKIRFESLTDKSKLD | 57 | - |
| motif 5 | ELIPEYLGFVKGIVDSDDLPLNISREMLQQNKILKVIRKNLVKKCLDMFFEIAE | 54 | HSP90 |
| motif 6 | QPELFIRIIPDKANGTLSIIDSGIGMTKADLVBNLGTIARSGTKEFM | 47 | HATPase_c |
| motif 7 | VSMIGQFGVGFYSAYLVAEKVIVTTKHNDDEQYVWESQAGGSFTVTRDTSGENLGRGTKITLYLKEDQLEYLEERRLKDLIKKHSEFISYPIYLWVEKTI | 100 | HSP90 |
| motif 8 | EDYNKFYERFSKNLKLGJSED | 21 | HSP90 |
| motif 9 | NKKPNNIKLYVRRVFISDBF | 20 | HSP90 |
| motif 10 | EINPEHPIIEELRKRADADPNDKSVKDLV | 29 | HSP90 |
